# On the Proper Selection of a Site for a Tuberculosis Sanatorium in the State of Texas

**Published:** 1907-04

**Authors:** C. H. Wilkinson

**Affiliations:** Galveston, Texas


For Texas Medical Journal.

### On the Proper Selection of a Site for a Tuberculosis Sanatorium in the State of Texas.

BY C. H. WILKINSON, M. D., GALVESTON, TEXAS,
Recent Manager of Camp Reliance near Comfort, Texas.

*Editor Texas Medical Journal:*

In view of the consideration of the subject announced at the head of this article, now going on by the lawmakers of our State and by other interested parties, and on account of the extreme importance of the question under consideration, I desire to offer some of the fruits of my experience bearing upon the subject, with the sole aim in view of aiding those who may soon be called upon to determine where a tubercular sanatorium in Texas should be located.

That our great and progressive State should construct and operate an institution of this kind for the benefit of its afflicted, is beyond any question of doubt.

The writer several years ago advocated the importance of this greatly needed charity, and the necessity for the institution is as urgent today as it was then, seven years ago.

Texas should own and operate a sanatorium for her tubercular citizens and such ownership and maintenance on her part should be inaugurated at as early a day as practicable.

The indications, however, seem favorable to the establishment of such needed institution at no very distant day, and hence the advisability of offering now the following suggestions upon the subject under consideration.

Of all the States in the Union none can offer more desirable locations for the establishment of a tubercular sanatorium than can Texas. In our great Empire State we can furnish almost any desirable climate that the world possesses, and as for altitude, we can supply any degree of elevation that the most exacting invalid could ask for. With almost perpetual summer for the entire year round, with cool, dry, bracing and refreshing breezes twelve months in the year, and with cloudless skies for most of the time, with an abundance of pure and sparkling waters gushing from gurgling springs, we can boast of an ideal land in certain portions of our State compared to which there are few more favored spots for the healing of tubercular cases in any quarter of the world.

Not all, but most of the territory lying between the Colorado and the Rio Grande rivers, and south of the thirty-first degree of latitude, might properly be called a natural sanitarium. In some of this territory fresh meat exposed to the direct rays of the sun in the open air, will remain pure and untainted for many months. No pus germs can survive in this climate, and suppurating wounds are never seen.

One can make his bed upon the lap of nature at night with only the starry canopy above him and sleep to his comfort and advantage all night long.

In selecting a location for a tubercular sanatorium, however, there are certain cardinal principles to be observed, a neglect of which will affect the utility of the institution established in proportion to the number of such principles neglected.

First, and above all things else, the atmospheric conditions abounding in such locality should be as nearly pure as possible. The air should be at all times free from dust, fog, and miasmatic contamination. It would be an unfortunate investment to locate a sanatorium along some stream which now and then sent forth its poisonous emanations in the shape of malaria. Yet there are just such objectionable localities to be found in the section of Texas I have just alluded to; localities which, otherwise, would have been ideal spots for establishing a sanatorium.

Second. Institutions of this kind should be located with a view to their accessibility by the invalid. Nearness to base has many advantages besides the facility of being reached by the broken-down health seekers. Supplies and help should be easily obtainable, and often they are needed very hurriedly, and this remark applies particularly to ice, vegetables and other necessities not advisable to mention.

If practicable a sanatorium should be located near thrifty towns or cities, for the simple reason that invalids often wish to visit such places for the purpose of relieving them of that terrible loneliness and ennui which hang so heavily over the spirits of the tubercular patient when away from home.

Consumptives require an occasional glimpse of civilization for the benefit of their health, and all sanatoria should be located with the view of giving them the benefit of this glimpse. They should be located on or near a railroad.

Third. Shade is another factor which enters largely into the list of essentials that are required to make a sanatorium successful. This shade should be made by the spreading branches of unbrageous trees and not alone by artificial awnings. Besides this, trees present a cheerful aspect to the invalid, and such invalids require all the cheerfulness that art and nature can afford them.

Too much stress can not be laid upon the importance of shade and an abundance of it in carrying out the cure of all tubercular cases.

Fourth. In whatever locality a tubercular sanatorium might be located, care should be taken that the soil abounding in that vicinity is rich and easily cultivated.

In Texas—should a sanatorium be agreed upon by our legislators, and eventually it will—it is more than likely that an industrial department could be operated in connection with the institution. That is to say, it would probably be found advantageous to offer if not insist on the abler class of invalids undertaking work of some description for the purpose of improving their condition. Moderate exercise is very beneficial to consumptives who are able to undertake it, and there is usually a large proportion of such invalids found in tubercular congregations. Exercise, especially when remunerative, is a mental diversion as well as a curative factor in such cases and is not to be underestimated in their treatment. It is probably safe to say that a large proportion of the cases who die from tuberculosis perish through physical and mental inertia. They should work when they are able; they should hustle when they can. Light work, and especially tilling of the soil, should be insisted on, and hence the necessity for having a sanatorium located where the soil could readily be cultivated.

As for elevation, it was formerly thought that the higher a consumptive ascended above the sea level, the better were his chances for recovery. This, however, is an error, and thousands of human beings have been sacrificed through ignorance on this subject. It is not essential to cure for a case to be located thousands of feet above the sea. Indeed, it often happens that tubercular cases improve by bringing them from a high to a low or to an intermediary elevation. This statement was often verified while the writer was conducting Camp Reliance at Comfort, for the treatment of tubercular cases, a few years ago. There patients came from Colorado and other elevated places much to their improvement, and I was importuned by one of them to raise my voice against the practice of sending advanced cases to such altitudes as Denver.

There are four degrees of elevation in Texas where consumptives can be placed to their advantage. These are the *high*, the *low*, the *high intermediary*, and the *low intermediary*.

The first would comprise all altitudes about 3000 feet and over. The low, an elevation running from sea level to about 300 feet above. The low intermediary might be placed between 500 and a thousand feet above the sea, while the last, or high intermediary, all that elevation between 1200 and 2000 feet in height.

There are numerous sub-varieties of most of these degrees, but they possess no practical value, and every one of these varieties can be found in Western Texas. Thus in Jeff Davis and El Paso counties we can find examples of the high; along the coast from Corpus Christi to the I. & G. N. railroad and thence west to the Rio Grande, and we have the low. All along the S. P. railroad, from Seguin to Del Rio, we have the low intermediary; while around Kerrville, San Angelo and Llano can be found the higher intermediary elevations. It is probably safe to say that for the purpose of locating a tubercular sanatorium—at any rate in Texas —the intermediary elevations are the preferable ones to select from. Either of these is desirable and eminently suitable as a locating place for a sanatorium, but the particular spot in any of this territory that can furnish the *essentials* alluded to before, that is pure air, shade, good water and accessibility, is to be preferred to any other, although in elevation it may not come up to the ideal laid down by writers on this subject. It is the *tout ensemble* of these conditions that constitute the preferable spot for a tubercular sanatorium, and in locating such an institution for our consumptives this ensemble should be properly observed.

Such then is a brief epitome of the points to be observed in the locating of a sanatorium for our tubercular patients. Located with due regad to all these features, such a place would prove successful beyond our highest expectations. A disregard of almost any of them would be followed with regret. The writer has made this subject a special study for the past ten years, and during more than half this time he has been abroad in Western Texas personally and practically investigating for himself. He has lived in Camp and Sanatoria with his tubercular cases for years, and he knows their requirements and their wants. He likewise knows what are the essentials to a successful sanatorium for consumptives, and hence the writing of this article.

Much could be said upon the proper construction of buildings and other quarters for consumptives, as upon this point hinges the very existence of such institutions, and upon their administration, so important in rendering them successful; but these topics will be deferred at present and until the proper time arises for their consideration, which will be after the location of the Texas Sanatorium for Consumptives has been determined on.

